# GANana: Unsupervised Domain Adaptation for Volumetric Regression of Fruit

**DOI:** 10.34133/2021/9874597

**Published:** 2021-10-08

**Authors:** Zane K. J. Hartley, Aaron S. Jackson, Michael Pound, Andrew P. French

**Affiliations:** ^1^School of Computer Science, University of Nottingham, NG7 1BB, UK; ^2^School of Biosciences, University of Nottingham, LE12 5RD, UK

## Abstract

3D reconstruction of fruit is important as a key component of fruit grading and an important part of many size estimation pipelines. Like many computer vision challenges, the 3D reconstruction task suffers from a lack of readily available training data in most domains, with methods typically depending on large datasets of high-quality image-model pairs. In this paper, we propose an unsupervised domain-adaptation approach to 3D reconstruction where labelled images *only* exist in our source synthetic domain, and training is supplemented with different *unlabelled* datasets from the target real domain. We approach the problem of 3D reconstruction using volumetric regression and produce a training set of 25,000 pairs of images and volumes using hand-crafted 3D models of bananas rendered in a 3D modelling environment (Blender). Each image is then enhanced by a GAN to more closely match the domain of photographs of real images by introducing a volumetric consistency loss, improving performance of 3D reconstruction on real images. Our solution harnesses the cost benefits of synthetic data while still maintaining good performance on real world images. We focus this work on the task of 3D banana reconstruction from a single image, representing a common task in plant phenotyping, but this approach is general and may be adapted to any 3D reconstruction task including other plant species and organs.

## 1. Introduction

3D reconstruction, the extraction of 3-dimensional shape information from one or more images, is commonly used as a high-throughput phenotyping technique. 3D information allows for simultaneous measurement of a variety of phenotypic traits. 3D fruit models in particular are useful for size estimation and quality control as well as assisting with precision breeding of different crops. Accurate measures of fruit volume can provide key traits for breeders and researchers, and this data can form part of a pipeline for other phenotyping tasks.

While there has been significant interest in applying different reconstruction methodologies to fruits in recent years, many of these methods involve the use of expensive hardware setups such as laser scanners, LIDAR, or multicamera setups to capture 3D structure. We focus here on the task of *monocular* reconstruction, the recovery of 3D structure from a single 2D image. One strength of our method is that it allows for accurate 3D reconstruction using only a single uncalibrated camera, making it easy to use and removing the prohibitive costs of more expensive setups.

For this project, we demonstrate the efficacy of our approach on bananas, chosen because they present a challenging variety of both 3D shape and colour and texture; for example, they are asymmetric and exhibit bruising and other unique texture features. Our subject choice also differs from other reconstruction methods that attempt to match a number of known key points to the target object, allowing our chosen method to be more generalizable to different domains. There is good availability of representative 3D models and photographs of bananas that may be used to produce synthetic and real datasets, aiding our domain adaptation approach that exploits both real and simulated data.

Like many other computer vision problems, large training datasets are needed when using deep learning for 3D reconstruction. Unlike common problems such as object detection or segmentation, 3D annotations are either impossible to create or very difficult to annotate, instead requiring additional data to be captured using specialised tools. It therefore quickly becomes expensive to produce training datasets, particularly at scale, outside of the most common problem spaces such as human pose or road features and vehicles.

3D reconstruction is, however, an important task in many areas and has been applied to fields including medical imaging, 3D mapping of human faces and bodies [1, 2], simultaneous localisation and mapping (SLAM) [3] for use in autonomous vehicles and augmented reality, and mapping the shape of various common objects for use in virtual environments [4]. Obtaining high-quality models can be difficult, and this 3D geometry may be encoded in many different ways, such as point clouds [5], 3D meshes, and voxel representations [6].

This difficulty in capture and a lack of cohesion between datasets make training from limited data a key challenge. This paper specifically focuses on a monocular approach via a volumetric regression network, lowering the cost and complexity of performing accurate 3D reconstruction, while our approach remains applicable across a wide number of domains.

In this paper, we propose a novel framework for training Deep Convolutional Neural Networks to accurately reconstruct 3D volumes of fruit and achieve a high level of accuracy while removing the hurdle of expensive data collection. Our approach frames the problem as one of unsupervised domain adaptation, using synthetic data from 3D modelling to avoid the difficult task of collecting ground truth 3D models with corresponding photographs of real bananas. Our model has two goals, first to transfer images from a *synthetic* domain to a *real* domain, while preserving the 3D geometry of the object in the image, and second to extract a volume of the object from the image. Unlike other works, which treat these as separate problems, our architecture is trained in an end-to-end fashion and is designed to be applicable to the widest variety of subject matter.

### 1.1. Motivations

Our experiments were motivated by the aim of greatly reducing the cost of solving monocular 3D reconstruction problems using deep learning, where hand annotation is not feasible and existing training datasets are scarce. Plant phenotyping comprises a wide variety of image subjects upon which phenotyping methods are applied. This makes problems of limited training data especially acute, so the field benefits greatly from methods such as ours that overcome this data scarcity problem.

A goal of our method was to make use of extensive libraries of 3D models now freely available from online sources. Leveraging this new source of data for deep learning is a promising solution to the data scarcity problem. Photo realistic models created for use in film, video games, and other renders can then be reused for any number of computer vision tasks. In our experiments, we have applied our method to the 3D reconstruction of bananas; however, our approach includes no domain-specific design choices and could be applied to any number of different objects so long as a number of accurate 3D models of a particular subject were available.

**Figure 1:**
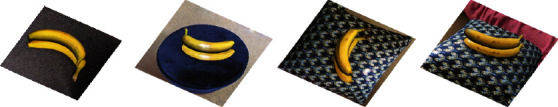
Results of our volumetric regression network. Output volumetric banana models resting on 2D input images.

In summary, our main contributions are as follows:

1. We demonstrate a novel architecture for unsupervised domain adaptation and 3D reconstruction from single views. Our approach is low cost, avoiding expensive acquisition of real 3D scans
2. We show that good performance can be achieved on 3D reconstruction of real images using only synthetic volumes, examples of our output can be seen in Figure 1
3. We release all code used in our pipeline, including scripts for the creation of our synthetic renderings through to training a volumetric regression network (VRN) with our created datasets
4. Finally, we make available our dataset of 25,000 synthetic banana images and their matching ground truth volumes on our project website

This paper will begin by giving an overview of closely related work in Section 2 before describing the materials and methods in detail in Section 3. We present our results in Section 4 and give an analysis of our results as well as discuss limitations in Section 5.

## 2. Related Work

This section examines related works in the fields of volumetric regression, generative adversarial networks, and domain adaptation of synthetic images.

### 2.1. 3D Reconstruction

While a full review of all literature on 3D reconstruction is beyond the scope of this paper, it is worthwhile noting a few popular methods. Application is wide and varied, for example, 3D reconstruction of blood vessels [7], multiview building reconstruction [4], face reconstruction [8], and view synthesis [9]. In particular, this work builds upon previous work for 3D face and body reconstruction using volumetric regression [1, 6], in which the shape of the object is encoded using voxels directly output by a deep network. Volumetric regression constrains the problem to a single domain, where both input and output are spatial, avoiding the need to learn a mapping from image to Euclidean or some PCA space. Volumetric regression has since been extended and refined to work more reliably on general human poses, such as in PIFuHD [2]. PIFuHD also demonstrates good performance at estimating 3D geometry for the nonvisible parts of the body.

### 2.2. Plant Phenotyping

Phenotyping refers to a collection of tasks that accurately measure quantifiable traits of plants. Being able to efficiently measure plant traits at scale aids the development of new crops and agricultural techniques; this has gained importance given both the climate crisis and the increasing global population. Image-based measurement of plant traits has become ubiquitous, helping with understanding environmental impacts on plants, as well as aiding breeding programs and production of crops. Some important works on plant phenotyping include the prediction of plant stresses [10], detection and segmentation of plants from aerial photography [11], and leaf or plant organ counting [12].

3D reconstruction of plant matter is important in solving a number of core tasks including growth measurement and yield estimation such as seen in work by Moonrinta et al. [13]. Jadhav et al. also use 3D reconstruction to help with the grading of fruit, with emphasis put on the importance of accurate reconstruction of arbitrary shapes [14]. Similarly, 3D reconstruction has been used to map the geometry of plant shoots, another common phenotyping task [15].

We are not aware of any methods which attempt to do 3D reconstruction from a single 2D image in the plant phenotyping space. Monocular approaches such as structure from motion have been used such as in Jay et al. [16]; however, these approaches require a sequence of frames instead of a single image as we use in our work. Beyond traditional RGB images, Wang and Chen [5] demonstrate fruit reconstruction using a Kinect sensor, while Feldmann et al. [17] perform shape estimation in strawberries using a turntable system to capture multiple images of a strawberry rotating on a calibrated spindle. Yamamoto et al. use an RGB-depth camera to generate 3D point clouds of apples by combining depth and RGB data [18]. Finally, Paulus reviews a number of works that use different laser scanning devices to capture point clouds for plant phenotyping [19].

### 2.3. Generative Adversarial Networks

Generative adversarial networks (GANs) are a form of deep learning in which competing networks are trained together. Although applications vary, GANs are commonly used to generate images. The original GAN framework included a generator which created new images from random noise and a discriminator which learned to distinguish images from a training set and those produced by the generator [20]. Since then, this framework has been adapted to many new problems, such as image generation [21], image to image translation [22, 23], and unsupervised recognition tasks [24]. In particular, DCGAN [21] is a popular model used for generating high-resolution realistic images from noise. DCGAN works across multiple domains, showing results for generation of both faces and bedrooms.

Conditional GANs, such as Pix2Pix [22], instead learn to produce images between two specific domains, such as the generation of city scenes from corresponding segmentation masks. Similarly, CycleGAN [23] also allows for domain transfer between both the target and source domains using unpaired images. CycleGAN uses a pair of generators and discriminators, which transform images between source and target domains in a *cyclic* manner. By ensuring images can be recreated in both directions, we ensure that an image's content is preserved while changing the image domain. We use CycleGAN as the backbone of our own architecture. More recently, SinGAN [25] demonstrated that a distribution can be learned from a single image and can be used for a number of varied image manipulation tasks such as harmonization and *paint-to-image*.

### 2.4. Domain Adaptation

Domain adaptation is a field of machine learning, related to transfer learning, that focuses on solving the domain shift problem in which a network trained to solve a task in one data distribution cannot generalize well on another similar distribution. A common benchmark for these problems is the popular character sets such as MNIST, USPS, and SVHN, which appear visually similar but are challenging for networks to generalize between [26]. More challenging tests include the office 31 dataset [27], which contains images of common objects from Amazon, webcam, and DSLR domains as well as VisDA [28] which focuses on simulation-to-reality shift for classification and segmentation tasks.

Unsupervised domain adaptation refers to problems where no labelled examples of the target domain are available [29]. In this case, a model must learn to make predictions based on the deep domain invariant features relevant to the task being solved. A number of recent approaches use CycleGAN-like models for pixel-level domain adaptation of synthetic images, similar to our own work. Mueller et al. introduced a geometric consistency loss to CycleGAN which focuses the generator on maintaining the same 2D geometry, while converting from the synthetic to real domain [30]. Their work differs from ours in that they separate the task of bridging the *synthetic to real* gap from the task they were trying to solve, whereas we combine the task in a single model that can be trained end to end. Mueller et al.'s work [30] is very similar to the work of [31] where they introduce the semantically consistent CycleGAN, which also uses segmentation masks to ensure the 2D shapes of different object are maintained by the generator; further examples can be seen in [32, 33].

The work of Shrivastava et al. shows a GAN model called SimGAN that uses a pairing of a refiner and discriminator to enhance images of synthetic eyes. Their method uses a self-regularization term to maintain gaze direction while enhancing synthetic data to photorealistic quality [34]. SimGAN was also used by Liu et al. and applied to the problem of human pose recognition and demonstrated state-of-the-art results [35].

## 3. Materials and Methods

In this section, we describe our approach to the problem of generating 3D volumes via unsupervised domain adaptation: in particular, how we crafted our datasets and selected the architecture of our model. In addition, we describe the experiments we conducted in order to test the efficacy of our proposed architecture.

### 3.1. Training Dataset

To train our model, we utilised two different datasets. The first is a collection of 25,000 images of synthetic bananas created in Blender [36] by rendering 5 *master* 3D banana models from freely available online sources (links to these will be provided on our project web page). Each model was chosen for its perceived realism, with more importance given to 3D geometry than to texture. These 5 models were then modified by scaling randomly along each axis to between 0.6 and 1.0 of their original size, followed by random in-plane rotation to create 5000 variations of each. We used the original provided textures for all captures of each master Banana; however, we adjusted the brightness of the light source between 0.5 and 1.5 times our default value, as well as adjusting some values of specular reflection to increase image variety. Renderings were captured of the augmented models, along with the random transformation parameters used.

The corresponding meshes were then used to create 3D volumes under the same transformations and were saved into an HDF5 file for input into PyTorch. For each rendering, a randomly selected image from the COCO dataset [37] was used as a background image, increasing variety in the training set and encouraging the generator to ignore the background. Augmentation and rendering were performed automatically in Blender, with volumetric ground truth produced in python. All required resources to adapt this pipeline to new datasets and domains will be released with this paper.

Our second dataset, consisting of real images, is a collection drawn from three sources. First, images were taken from the dataset [38] originally used for ripeness classification networks. Second, the “*Top Indian Fruits*” dataset contains many images of bananas in various states of ripeness and health [39]; from this, we selected only the examples of healthy bananas and discarded the associated per-image ripeness and quality labels. Finally, we collected additional images ourselves, allowing us to add images with more variations in lighting and angle. To further increase the variety in our dataset, these images were also augmented with scaling, flips, and rotations to generate 25,000 different examples.

### 3.2. Voxelisation Procedure

The rendering process saves the applied rotation and projection matrix with each banana rendering.

In order to bring the 3D model into alignment such that it may be voxelised, we first apply the rotation transformation, followed by projection transformation. The projection matrix destroys depth information in the *Z* axis with respect to the image plane. We recover this by using the standard deviation of the 2D axes, before and after the projection step, as a scaling factor for the *Z* axis. The standard deviation of *x* and *y* is used because it is invariant to any translation which may have been applied during projection. More concretely, where *M* and *M*_proj_ are the unprojected and projected meshes, respectively, of *x*, *y*, *z* coordinates,

(1) $$\begin{array}{c} M_{\text{proj},z}=\frac{M_{2}}{2}\left(\frac{\text{std}\left(M_{\text{proj},x}\right)}{\text{std}\left(M_{x}\right)}+\frac{\text{std}\left(M_{\text{proj},y}\right)}{\text{std}\left(M_{y}\right)}\right). \end{array}$$

Voxelisation is performed by tracing rays through each plane, *x*, *y*, and *z* to produce three intermediate volumes. These are combined into a single 3D volume by finding all voxels that intersect at least two of the intermediate volumes. This approach reduces artefacts but is slightly slower than performing voxelisation from a single plane (we use Adam Aitkenhead's implementation, available at http://uk.mathworks.com/matlabcentral/fileexchange/27390-mesh-voxelisation). Our final volumes have a resolution of 256 × 256 × 128.

Higher depth resolution is unnecessary in this problem domain.

### 3.3. Volumetrically Consistent CycleGAN

**Figure 2:**
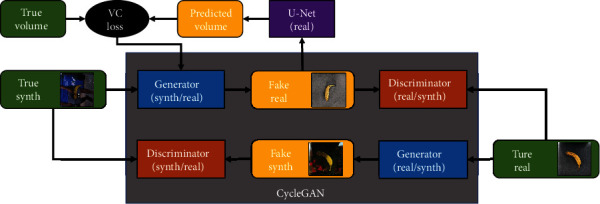
The proposed volumetrically consistent CycleGAN (VCC) using our *real* banana dataset as a target.

Our goal is to train our end-to-end network to produce a 3D reconstruction of objects from the images in the *real* domain. We extend a CycleGAN implementation [23], shown in Figure 2, to perform unpaired image-to-image translation between *real* and *synthetic* images.

Our novel addition here is a VRN that performs 3D reconstruction on the output of the *synthetic to real* generator. We evaluated a number of models for this task, including U-Net [40] and stacked hourglass models shown in [6], and found that a modified U-Net implementation achieved the best performance in early experiments. We use standard spatial convolutions throughout the network and reconfigure the U-Net to use three downsampling layers followed by three upsampling layers. Comparing this loss against the true volume of the synthetic image gives us our *volumetric consistency loss* (VC loss), for which we selected binary cross entropy (BCE). This loss is applied first to the generator, which ensures that the 3D structure of the object is preserved when changing the domain of the image and additionally the U-Net. The VC loss is given a weight of 1.0, relative to all CycleGAN weights which are given their default values. This value was determined empirically, though further fine tuning may improve time taken for convergence.

It has been shown that CNNs trained on purely synthetic data do not generalise well onto real images [6]. Large performance increases can be achieved by including a small fraction of real training data [41]. Our approach extends this idea by requiring only labelled synthetic data supplemented with different datasets of *unlabelled* real photographs as input.

### 3.4. Experiments

1. *VRN Trained with Synthetic Data Only*. Here, we establish a baseline in terms of performance, i.e., what level of performance we can achieve on real images when trained only on synthetic renders. Synthetic images have been successfully leveraged in many domains, but the domain gap between synthetic and real images often leads to poor generalisation.
2. *VRN Trained on CycleGAN Images*. We evaluate the performance of the VRN on real images, when synthetic training images have first been *refined* to look more realistic. CycleGAN is trained to translate the synthetic images into the target domain of real images which are then used to train our VRN as carried out in experiment 1.
3. *GANana VRN*. GANana combines the VRN and CycleGAN in a single model, shown in Figure 2, that can be trained end to end. Images are refined by the CycleGAN at the same time as our VRN is trained to extract a 3D volume. The approach taken by GANana ensures that refined images preserve the high level structural features necessary for volumetric reconstruction while simultaneously closing the domain gap between the two sets of images.
4. *GANana VRN using PASCAL VOC*. In this experiment, we use the same architecture from experiment 3 but replace our *real* banana dataset described in Section 3.1 with unlabelled images from the PASCAL VOC dataset. We hypothesised that a wider range of images from the *real* domain may compensate for using images that do not match the particular subject of our source domain and, if so, reduce the need to build a domain-specific dataset.
5. *GANana VRN using Gaussian Noise*. For this test, we replace our target domain dataset with random noise. We hypothesise that this will force our generator to transform our image almost entirely into noise, maintaining only the high level features needed to regress the banana. By excluding images from the target domain, we prevent the model from performing domain adaptation, and any improvement on our baseline score can be attributed to augmentation. Unlike our previous experiments, in this example, losses from the VRN and CycleGAN will, we hypothesise, be sufficiently opposed to each other such that it will be impossible to produce good results.
6. *GANana VRN using Synthetic Target*. In our final experiments, we train on pairs of *identical* images from our synthetic dataset as both the source and the target domain. By keeping the source and target domains the same, CycleGAN is no longer encouraged to transform input images, as any transformation made by the generator can only make each image differ from the target. Instead, we hypothesise that it will apply subtle augmentations to each image, improving robustness of our VRN while being prevented from significantly altering the high level features of each image. Increased variability of the input data means the VRN in our model must be more resilient to augmentations produced by the generator, which may enable it to perform well on images in our target domain. In this sense, we can consider the goals of our CycleGAN and VRN to be better aligned, which we believe will improve performance.

### 3.5. Testing Dataset

**Figure 3:**
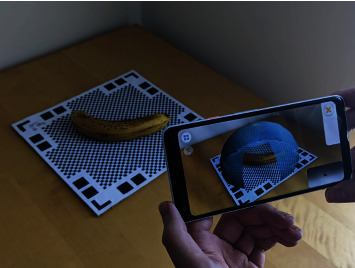
Demonstration of capturing instances for our test dataset through the Qlone photogrammetry mobile app.

In order to test our method, we built our own test dataset comprising 15 real banana models with associated 3D ground truth. Images were captured using the photogrammetry app Qlone, run on an Android phone [42]. For each model, a banana was placed on a calibration base and images were captured from numerous angles. The banana was then flipped onto a different side and the process was repeated to improve accuracy on the unseen surface.  Figure 3 shows this process. The app combines the two meshes to generate a single 3D model of the banana for import into Blender, where any element's remaining errors such as reconstructed background could be removed manually. The process described in Section 3.2 was used to convert each model into a volume for use as ground truth. Finally, each model was paired with a single top-down image of the banana it was generated from, which would then make up each test image-volume pair. Each example took an average of 15 minutes to capture, demonstrating the difficulty in feasibly collecting enough samples to create a suitable size dataset for training a VRN with real image-volume pairs, as has been demonstrated in previous works [6].

### 3.6. Training

Our network was trained in an end-to-end fashion using the Adam optimizer, a learning rate of 2*e* − 4, and default parameters for all CycleGAN models used in the architecture. We trained the model using a batch size of eight and trained on eight NVIDIA Titan X (Pascal) graphics cards for 10 epochs until the model converged. In order to decrease training time when loading our training data, we saved our dataset in HDF5 format, allowing it to be directly loaded as a PyTorch Tensor. We perform limited online augmentations to both images and volumes, including flips and 90- and 180-degree rotations in order to ensure our network generalises well onto a wide range of test image examples.

## 4. Results

Here, we present the results of the experiments conducted to evaluate the effectiveness of the model described in Section 3.

### 4.1. Qualitative Results

**Figure 4:**
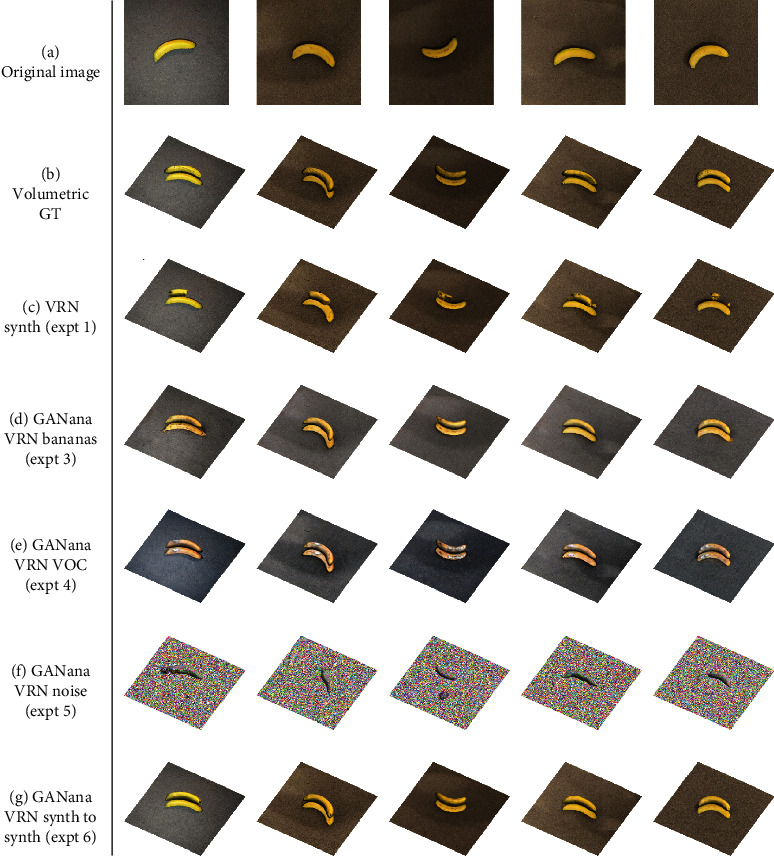
Example outputs of our experiments for volumetric regression showing ground truths (b), synthetic training (c), and then our GANana models with different target datasets (d–g).

**Figure 5:**
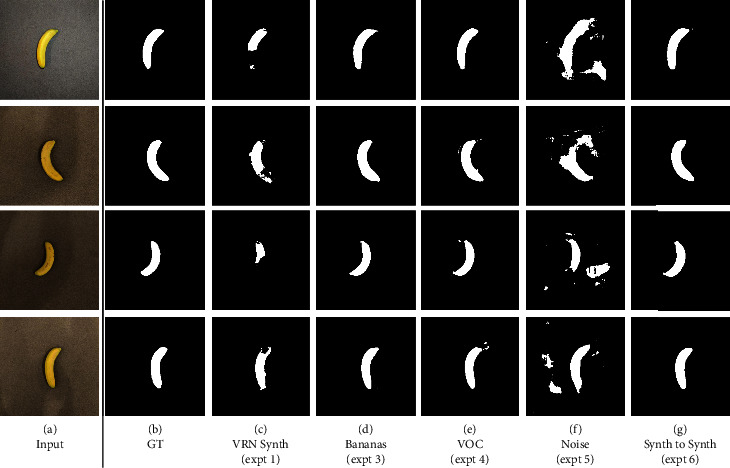
Outputs of our experiments for 2D segmentation showing ground truth segmentation masks (b), synthetic training (c), and our GANana models (d–g).

We show the input with corresponding output, from the four experiments, in Figures 4 and 5. VRN trained on only synthetic images (experiment 1) fails almost completely when presented with a real image. GANana succeeds in cases (3), (4), and (6), with only the addition of unlabelled target images. The background images in Figures 4(b) and 4(c) are from the original images, but in Figures 4(d)–4(g), the 2D image output from the *synth-to-real* generator component is used as a background, which gives an idea of how the generator transforms input images depending on the target dataset used in each experiment. These images demonstrate that the volumetric consistency prevents distortions to the original object's shape and that the main difference from the transformation appears to be colour tone.

**Figure 6:**
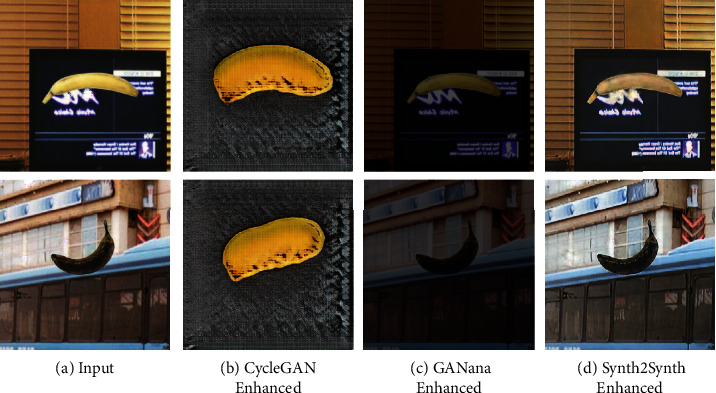
Output from *synthetic to real* generator from standard CycleGAN (b), GANana with volumetric support (c), and CycleGAN with synthetic as target (d).

In Figure 6, we show the output of the generator both *with* (Figures 6(c) and 6(d)) and *without* (Figure 6(b)) the proposed volumetric consistency loss. CycleGAN is known to have a number of failure cases, especially where the two training domains are not sufficiently similar [23], and we see an example of this in experiment 2. Without the volumetric consistency loss, the model degenerates to creating very similar images that do not retain their structure, hardly resembling a banana at all; and as such, we have not included it in our results in Table 1. This fail state is consistent with what is observed in [30], where CycleGAN is unable to preserve geometry when transforming an image from the synthetic to the real domain, and Mueller et al. are able to improve augmentation by using a 2D segmentation network to provide a support loss in order to generate images for hand tracking. We speculate that 3D renders and photographs of real bananas are not sufficiently similar for CycleGAN to produce good results; it is a strength of our model that it performs well even where these higher-level differences between our two datasets exist. As evidence of this, we observe the GANana-enhanced images exhibit contrast and brightness changes that better match images from the target domain. As such, CycleGAN-learned transformations are more pronounced on images which differ more significantly from those in the target set, while appearing less extreme on more similar images as we observe in Figure 6.

### 4.2. Quantitative Results

For each experiment, we compute both Volumetric Intersection over Union (VIoU), as well as Root Square Mean Error (RSME). To compute both metrics, we accounted for scale using the length, width, and depth of each banana, before applying the Iterative Closest Point (ICP) algorithm. This procedure was repeated three times for each sample, which we found to produce adequate alignment to obtain the best mapping between reconstruction and ground truth and avoid simple translation and rotation errors. ICP was needed as the scans produced by the Qlone app were scaled differently to the predicted 3D volume and not aligned with the individual photo. Our results are therefore presented after ICP alignment. This may bias the performance slightly, but the same procedure was used for all experiments for consistency.

We present our numerical results in Table 1. The baseline VRN trained with synthetic data (1) performed very poorly on real images. This is likely due to the domain gap between real and synthetic images causing poor generalization between images which may on first impressions appear visually similar. Conversely, in experiments 3 and 4, using our volumetrically consistent GAN, we are able to improve performance substantially, and both experiments achieve our highest VIoU scores. As predicted, experiment 5 shows a marked decrease in performance compared to our other experiments using our architecture especially in 2D IoU but still outperforms our baseline score, despite images being almost completely indistinguishable from noise. Experiment 6, however, performs well and has scores that are comparable to experiments 3 and 4 and significantly above the baseline. This is an interesting and significant result, as these scores are achieved when testing on real images despite being trained with only synthetic images, and does not require a dataset of even general real images as a target.

### 4.3. Segmentation

Here, we demonstrate that our method is capable of performing 2D segmentation. By taking the sum of the produced volumes through the *Z* axis, we predict a segmentation mask, to enable comparison with a silhouette from the source 2D image. We had annotated foreground and background pixels for images in our testing dataset. In the second column of Table 1, we show Intersection over Union score, demonstrating that our method is also effective at training for 2D segmentation through domain adaptation, as well as measuring shape error as viewed from directly above.

### 4.4. Method Performance

Methods working with volumetric structures have a reputation for being either slow or inefficient. The volumes themselves are often large and can be difficult to work with. However, binary volumes which have large contiguous blocks of data (such as ours) are highly compressible. Our 256 × 256 × 128 volumes are stored as one byte per voxel, thus requiring 8 MB of memory per volume. However, on disk with LZ4 compression, they consume only 70 kB to 90 kB with minimal computational cost.

Our architecture contains no 3D (volumetric) convolutions and instead uses only 2D (spatial) convolutions, which are highly optimised to run on the GPU. Inference through our model takes 253 ms on a single NVIDIA Titan X (pascal). This is then followed by an additional 124 ms to extract the surface from this volume (allocated a single core on an Intel Xeon E5-2698 v3 system, average for 1000 runs).

We believe that for many applications, the ability to create a 3D model in under 400 ms from a single image is practical. Further improvements are likely possible, too.

## 5. Discussion

In this section, we present an analysis of our results and the effectiveness of our methodology as demonstrated by our experimentation. We also talk about some possible limitations of our method and possible improvements or additions that could be made to our pipeline.

### 5.1. Analysis of Results

The small differences in performance between experiments 3 and 4 are sufficiently small such that both results can be considered equivalent and, in both cases, show good performance in both 2D and 3D. It is interesting that domain-specific target images are not shown to lead to substantial performance increases and show the broader applicability of our architecture. Experiment 3 produces the highest score for 2D IoU, suggesting that using real bananas as our target domain encourages the generator to best maintain the outline of the banana during transformation. Experiment 4 shows that targeting PASCAL VOC with the GAN achieves comparable results in terms of VIoU 374 and RMSE, compared to using our *real* banana dataset. This is significant as it demonstrates that our method is effective even if large datasets of the particular subject matter are unavailable. We believe this demonstrates our method's potential to work in other domains.

In experiment 5, the VRN is still able to extract a reasonable likeness to the true volume, suggesting that structural information must still exist in the noise images in order allow reconstruction of the volume via the VRN. As the VRN is trained on images which have been passed through the CycleGAN with noise as a target, the loss of the VRN encourages preservation of high level features that enable the regression of the volumetric structure. Experiment 6 performs well given it is trained exclusively on synthetic images; however, we believe that the performance benefit obtained by using a real target dataset as seen in experiments 3 and 4 is worth the small additional cost of curating a selection of real images, particularly when they can be easily sampled from existing datasets. Aside from transforming images from one domain to another, it is conceivable that CycleGAN is simply performing image augmentation, thus treating the task as a domain generalisation problem and forcing our VRN to be robust to variation. The fact that experiment 6 performs comparably to experiments 3 and 4 without using images from the target domain would support this hypothesis.

### 5.2. Limitations and Failure States

In Figures 4, we see that in experiment 5, there are a number of fail cases, where background pixels are interpreted as part of the volume by the VRN, and this leads to even poorer performance for 2D results as shown in Figure 5. These kinds of false positive results are not observed in other experiments or even the baseline; we hypothesise that this is caused by the *noise* target domain having no distinction between foreground and background pixels for the network to learn.

Although in our other experiments, our GANana VRN models performed well on our test dataset, it is likely our approach has limitations in its effectiveness that may lead to failure states. Because our training is based on automatically generated synthetic data, it makes it more likely that failure states will emerge when images sufficiently different from the training set are tested. An example observed during testing was a failure state when the banana is not well centred in the frame, as they are in our synthetic models.

Similarly, although controlled-light phenotyping tasks are common, in other phenotyping tasks, it is possible for extreme lighting situations to present a challenge. However, while our testing was carried out with controlled lighting, it is likely our networks will be more resilient to these kinds of changes as CycleGAN has the potential to improve the VRN's ability to generalise onto more varied images.

### 5.3. Future Work

In our work, we focus on accurate 3D reconstruction of fruit using a methodology that has not yet been applied in a real-world phenotyping pipeline; as such, we do not calibrate our images to real-world units. We believe that by capturing images using a well-calibrated capture environment, it would be possible to estimate both the volume and the mass of fruit using an extension of our proposed setup.

## 6. Conclusion

We have presented our methodology for using a VRN trained on augmented synthetic data to address the problem of estimating accurate 3D models from a single view. These models, trained on a fruit dataset, provide detailed 3D reconstructions of the target object, ideally suited to downstream phenotyping tasks. Our results are obtained with a smaller data and annotation cost than conventional deep learning models by approaching the task as an unsupervised domain adaptation problem. As such, our approach provides full reconstruction of the target object without the need for any manually annotated real-world images. We introduce a volumetrically consistent CycleGAN, in which a CycleGAN is used to transform an image from a labelled synthetic domain into an unlabelled real domain, while a volumetric regression network learns to reconstruct object models in 3D. These networks are trained end to end, improving performance over a modular design. We have shown a significant improvement in volumetric segmentation scores and RMSE versus alternative approaches. Our approach performs well against ground truth generated using multi-image photogrammetry software and demonstrates our model's ability to generate accurate reconstructions.

This accurate reconstruction of 3D models of plants is important in the push for automated size and quality control, as well as other phenotyping tasks such as informing biological modelling applications. Common hardware-based techniques such as LiDAR are costly and time-consuming, unsuitable for very high-throughput pipelines. Our method is fast (*<*0.5 seconds per image) and accurate, requires no human interaction once trained, and works using a single RGB camera. We expect that the method concept will generalise to a wide range of other objects, including other fruit, vegetables, and plant organs such as leaves. To apply this technique to new domains, the production of appropriate synthetic models is required, combined with sample images from the real domain. Our software pipeline, dataset, and network will be made available online, to facilitate researchers training 3D reconstruction models in a variety of domains.

## Figures and Tables

**Table 1:** Volumetric IoU and RMSE reported on our collected dataset of real bananas.

Method	2D IoU	VIoU	RMSE
(1) VRN (synth training)	41.74%	17.52%	7.59
(3) GANana (bananas)	92.36%	76.37%	1.68
(4) GANana (VOC)	91.88%	76.60%	1.65
(5) GANana (noise)	44.65%	33.04%	7.64
(6) GANana (synth to synth)	92.29%	73.62%	2.07

## Data Availability

The code used to create the dataset for this study has been deposited on github at https://github.com/zanehartley. The code for the neural networks used for this study has been deposited on github at https://github.com/zanehartley. The image-volume datasets used for this study have been deposited at https://plantimages.nottingham.ac.uk/.
